# Gene delivery in mouse auditory brainstem and hindbrain using *in utero* electroporation

**DOI:** 10.1186/s13041-014-0051-4

**Published:** 2014-07-26

**Authors:** Laurence S David, Jamila Aitoubah, Lee Stephen Lesperance, Lu-Yang Wang

**Affiliations:** 1 The Hospital for Sick Children Research Institute & Department of Physiology, Program in Neurosciences and Mental Health, University of Toronto, 555 University Ave, Toronto M5G 1X8, ON, Canada

**Keywords:** In utero electroporation, Gene delivery, Krox20, Hindbrain, Calyx of Held, MNTB, GFP

## Abstract

**Background:**

Manipulation of gene expression via recombinant viral vectors and creation of transgenic knock-out/in animals has revolutionized our understanding of genes that play critical roles during neuronal development and pathophysiology of neurological disorders. Recently, target-specific genetic manipulations are made possible to perform in combination with specific *Cre*-lines, albeit costly, labor-intensive and time consuming. Thus, alternative methods of gene manipulations to address important biological questions are highly desirable. In this study, we utilized *in utero* electroporation technique which involves efficient delivery of hindbrain-specific enhancer/promoter construct, Krox20 into the third ventricle of live mouse embryo to investigate green fluorescent protein (GFP) expression pattern in mouse auditory brainstem and other hindbrain neurons.

**Results:**

We created a GFP/DNA construct containing a Krox20 B enhancer and β-globin promoter to drive GFP expression in the hindbrain via injection into the third ventricle of E12 to E13.5 mice. Electrical currents were applied directly to the embryonic hindbrain to allow DNA uptake into the cell. Confocal images were then acquired from fixed brain slices to analyze GFP expression in mouse whole brain at different postnatal stages (P6-P21). By using a cell-type specific enhancer as well as region specific injection and electroporation, robust GFP expression in the cerebellum and auditory brainstem but not in the forebrain was observed. GFP expression in calyx of Held terminals was more robust in <P15 compared to >P15 mice. In contrast, GFP expression in MNTB neurons was more prevalent in >P15 compared to <P15. In regards to the relative expression of GFP versus the synaptic marker Vglut1, percentage fluorescence GFP intensity in the calyx was higher in P11 to P15 than P6 to P10 and P16 to P21 groups.

**Conclusions:**

Taken together, this technique would potentially allow hindbrain-specific genetic manipulations such as knock-down, knock-in and rescue experiments to unravel critical molecular substrates underpinning functional and morphological remodeling of synapses as well as understanding the pathophysiology of certain neurological disorders targeting not only the auditory brainstem but also other parts of hindbrain, most notably the cerebellum.

## Background

Recent advances in understanding genetic mechanisms underlying normal neuronal development and pathophysiology of a number of nervous disorders have been made possible by gene manipulations using loss- and gain-of-function transgenic animal models via viral infection or *Cre*-Lox system. However, it is not always feasible to restrict or repress expression of genes spatially and temporally, and the generation of transgenic animals and recombinant viruses are costly, time-consuming and labor-intensive [[1]–[5]]. To address these issues, alternative and innovative method of gene delivery into the mouse developing embryo known as *in utero* electroporation has been developed. To date, in *utero* electroporation has been successfully used to study structures in cortex, retina, thalamus and hypothalamus [[3]–[5]] as well as in dendritogenesis and migration of developing cerebellar Purkinje cells [[6]]. However, there is no report yet on gene delivery on murine hindbrain, and specifically the auditory brainstem using the *in utero* electroporation technique.

The development of vertebrate hindbrain is determined by the formation of segmentation processes along the anteroposterior axis leading to the establishment of transversal morphological units called rhombomeres (r). The rhombomeres are structural units that direct cell lineage patterns and area of gene expression [[7]–[10]]. The molecular control of cell- and tissue-type specification within developing hindbrain provides insights into the genetic bases of the most common human developmental brainstem and cerebellar disorders such as congenital deafness, migraine, vestibulopathies, Joubert syndrome, Dandy-Walker malformation, spinal cerebellar ataxias (SCA) and many other hindbrain disorders. Indeed, several causative genes have been identified for cerebellar and brainstem disorders [[11]–[13]]. Thus, it is imperative to develop a method to efficiently deliver gene(s) into the hindbrain using both region-specific injection and incorporating a hindbrain-specific enhancer construct.

Krox20, a C_2_H_2_-type zinc finger transcription factor and immediate early gene, is highly specifically active in r3 and r5 of the developing hindbrain giving rise to majority of the brainstem and cerebellar neurons [[14]]. Krox20 mRNA is first detected in prospective r3 and r5 at approximately embryonic (E) 8 and 8.5, respectively. At E9.5, Krox20 mRNA expression begins to disappear in r3 and at E10.5 expression starts to disappear in r5. However, between E14.5 and E16.5, a second period of transcription was observed in two dorsolateral columns of brainstem [[15]–[17]]. In regards to cellular localization and developmental regulation of Krox20 protein expression, De et al. showed that Krox20 has prominent role towards the maturation of motoneurons and somatosensory hindbrain neurons, Purkinje cells and components of auditory brainstem [[18]]. Given the role of Krox20 in hindbrain development, we utilized the *cis*-regulatory element Krox20 B enhancer [[19]] to investigate the possibility of hindbrain-specific gene delivery. We cloned fluorescent labeled DNA construct that would drive expression into the mouse hindbrain. We incorporated Krox20 B enhancer upstream of β-globin promoter and enhance green fluorescent protein (EGFP) reporter to test the efficiency and specificity of delivering genes via in *utero* electroporation. Confocal images from fixed postnatal mouse brain slices were obtained and analyzed to evaluate competence of this gene delivery technique. Our results demonstrated that Krox20 can drive the expression of GFP in subsets of neurons in hindbrain, and hence provide a proof of principle that genes of interest can be targeted via *in utero* electroporation for molecular perturbations to facilitate understanding molecular underpinnings of cerebellum and brainstem during normal development and diseases.

## Results

### GFP expression patterns in hindbrain neurons

**Figure 1:**
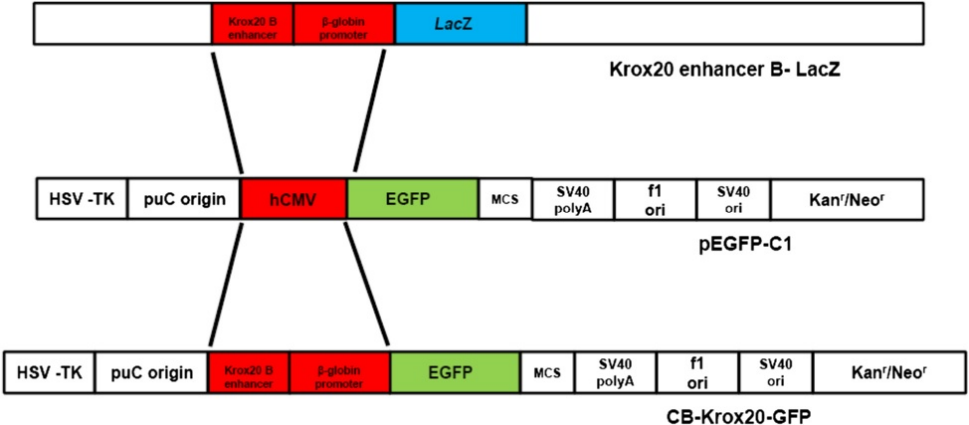
**Diagrammatic illustration depicting cloning strategy for Krox20 B enhancer/β-globin GFP construct utilized in this study.** The CMV promoter region of pEFGP-C1 vector (Clontech, Invitrogen) was excised and replaced with Krox20 B enhancer and β-globin promoter from cB-LacZ construct kindly donated by Dr. Pascale G-Heberstreit (INSERM, France) creating the CB-Krox20-GFP construct which was subsequently used for *in utero* injection and electroporation experiments.

**Figure 2:**
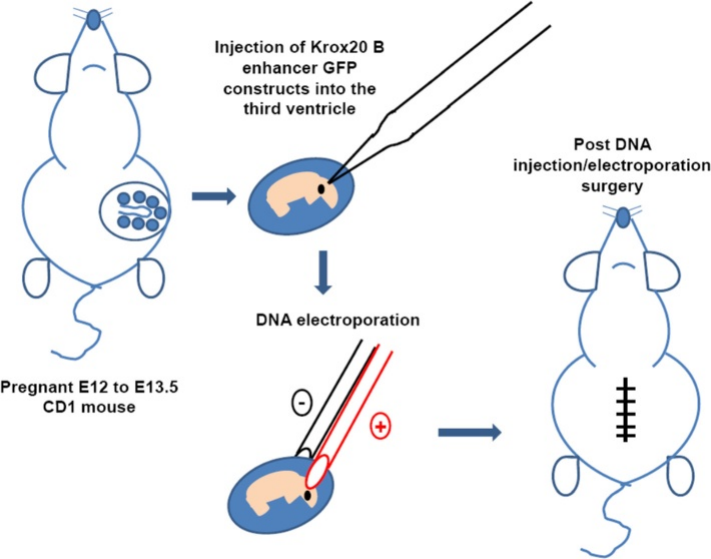
**Schematic illustration depicting*in utero*injection and electroporation of Krox20 B enhancer-GFP construct into the 3rd ventricle of E12-13.5 embryonic mouse brain.** Pregnant CD1 mouse (E12 to E13.5) was injected with CB-Krox20-GFP DNA construct or pCDNA3.1(−) (~1 to 2 μg) was injected to the lateral ventricle of mouse brain using glass capillary tip (Drummond Scientific, Broomall, PA, USA) directed towards the third ventricle to target the hindbrain. After DNA injection and electroporation embryos were carefully replaced back into the abdomen and the wound was closed. Detailed protocol is described in *Methods*.

**Figure 3:**
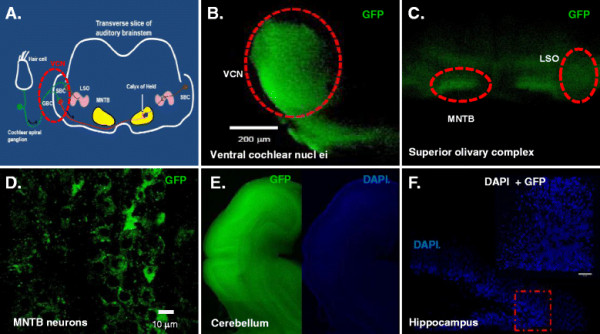
**Krox20 B enhancer directs GFP expression in auditory brainstem and other hindbrain neurons but not in the forebrain. (A)** Diagram showing components of auditory brainstem. Examples of GFP expression in **(B)** ventral cochlear nuclei (VCN), **(C)** lateral superior olive (LSO) and medial nucleus of the trapezoid body (MNTB) and **(E)** cerebellum. Absence of GFP expression was evident in **(F)** hippocampus. All images obtained from sagittal and transverse mouse brain slices. Images were acquired using 5X magnification except for **(D)** MNTB neurons and **(F)** hippocampus (63X) with a Zeiss LSM 510 Multiphoton Laser Scanning Microscope equipped with 405 and 488 lasers to visualize nuclei (DAPI) and GFP, respectively. 3D image of hippocampal neurons (**F** inset) was constructed using Volocity software (Perkin Elmer, USA). Scale bar is at 10 μm. Overlapping 5X images and 63X images were taken and compiled to generate composite images of cerebellum and hippocampus for both green (GFP) and blue (DAPI) channels to illustrate distribution of GFP expression in mouse brain. Image contrast enhancement was applied post acquisition for aesthetic reasons.

In order to assess the efficiency of targeting structures in the brainstem and cerebellum, both region- specific and enhancer/promoter-specific injection and electroporation were performed in this study. As described in *Methods*, Figures 1 and 2, GFP DNA construct containing Krox20 B enhancer and β-globin promoter was cloned and injected into the third ventricle of the brain. DNA was visualized using 0.05% trypan blue and allowed to enter to the third ventricle of the developing E12 to E13.5 embryo (Figure 2). The pattern of GFP neuronal expression was examined using brainstem and whole brain slices taken at different postnatal stages and visualized under laser scanning microscope. Postnatal pups from CB-Krox20-GFP electroporated mice showed GFP expression in the hindbrain particularly the auditory brainstem and cerebellum but not in the forebrain. We observed expression in ventral cochlear nuclei (VCN), lateral superior olive (LSO), and medial nuclei of the trapezoid body (MNTB) (Figure 3B, C and D) as well as in the cerebellum (Figure 3E). On the other hand, GFP expression was not evident in the forebrain such as in the hippocampus (Figure 3F) as revealed by absence of green fluorescence. To test for autofluorescence from the animals *per se* or from the technique, pups from mouse electroporated with pcDNA3.1 (−) were also analyzed. Our results showed absence of autofluorescence from the animals and procedure (data not shown).

### Developmental dependence of GFP expression in pre- and postsynaptic elements of synapses

The calyx of Held-principle neuron synapse in the medial nucleus of trapezoid body (MNTB) in the superior olivary complex (SOC) in auditory brainstem is an important relay station in the sound localization circuit, capable of high fidelity neurotransmission to project well-timed and sustained inhibition to other auditory nuclei. Because of its giant size and accessibility to patch-clamp recordings, this large axosomatic terminal at this synapse has emerged as one of the most prominent preparations for anatomists and physiologists to study important questions with respect to fundamental principles of developmental plasticity in presynaptic morphology, excitability and transmitter release [[20],[21]]. Aside from acute stereotactic surgery to inject viral constructs into postnatal mice synapse [[22],[23]], it has been difficult to perform molecular perturbations in order to study mechanisms underlying structural and functional remodeling in the developing calyx of Held-MNTB. Therefore, it is imperative to develop innovative methods that would allow genetic and molecular manipulations to target these synapses early in development. In this study, we explored the potential utility of *in utero* electroporation technique via analysis on the developmental progression of GFP expression from postnatal (P) pups electroporated with CB-Krox20-GFP DNA construct.

**Figure 4:**
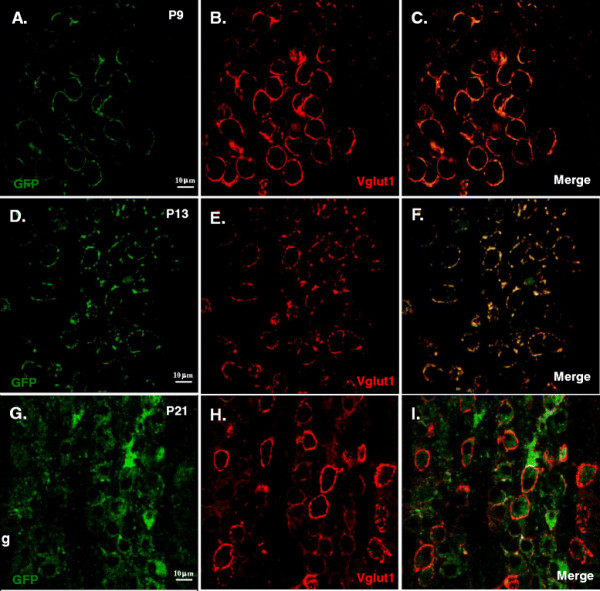
**Developmental dependence of GFP expression in calyx of Held – MNTB synapse.** Examples of images from postnatal pups taken from E12 – E13.5 *in utero* electroporated pregnant mouse injected with Krox20 B enhancer/β- globin promoter-GFP DNA construct. Brainstem slices from MNTB/calyx region from three age groups were analyzed. Transverse brainstem slices from P9 **(A, B and C)**, P13 **(D, E and F)** and P21 **(G, H and I)** mice were obtained and processed to determine the expression of GFP **(A, D and G)** using Vglut 1 **(B, E and H)** as presynaptic marker and overlay images are also shown **(C, F and I)**. Confocal images and z stack images were acquired as described in Methods. Image contrast enhancement was applied post acquisition for aesthetic reasons.

Three groups of electroporated postnatal pups were studied, P6 to P10, P11 to P15 and P16 to P21 representing pre-hearing, intermediate and post-hearing stages, respectively. Representative images showing developmental-dependence of GFP expression are summarized in Figure 4. Confocal images were obtained as described in *Methods* section. The vesicular glutamate transporter 1 (Vglut1) was used to stain calyces.

**Figure 5:**
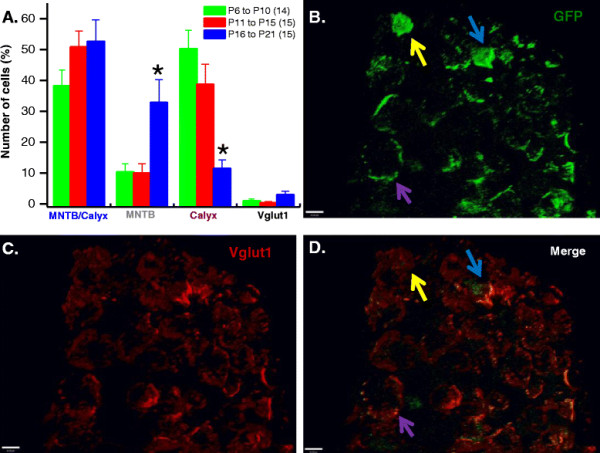
**Developmental switch in dominant expression of GFP from presynaptic to postsynaptic element. (A)** Quantitative analysis on the distribution of GFP expression driven by Krox20 B enhancer/β- globin promoter in the MNTB and calyx of Held synapses from three age groups. Three age groups were analyzed: P6 to P10 (pre-hearing); P11 to P15 (intermediate); and P16 to P21 (post-hearing). Presynaptic terminals expressing Vglut1, but not GFP were also quantified. In the calyx, GFP expression is more robust in P6 to P10 and P11 to P15 age groups compared to P16 to P21. On the other hand, in the MNTB neurons expression of GFP is more prevalent in P16 to P21 compared with the two younger age groups. Data represents mean ± s.e.m. Asterisks indicate significant difference at p < 0.05 (ANOVA) using ORIGIN 7.5 (Northampton, MA). N represents number of images from each age group from at least three animals. Examples of 3D images from the MNTB and calyx of Held synapses area: **(B)** GFP, **(C)** Vglut1 and **(D)** merge. Number of cells expressing GFP were counted as calyx only (purple arrow), MNTB only (yellow arrow) and both calyx and MNTB (blue arrow). Confocal and z stack images were acquired as described in *Methods* and 3D images were rendered using Volocity software (Perkin Elmer, USA). Image contrast enhancement was applied post acquisition for aesthetic reason.

The spatial distribution of GFP expression was evaluated by counting cells that expressed GFP in the calyx alone, MNTB alone and both in MNTB and calyx. Presynaptic terminals expressing Vglut1, but not GFP were also quantified. Figure 5 shows representative 3D images from the MNTB and calyx of Held synapses area confirming expression of GFP (Figure 5B) the synaptic marker, Vglut1 (Figure 5C) and superimposed GFP and Vglut1 (Figure 5D). In the calyx, percentage GFP expression (Figure 5A) was more robust in P6 to P10 (50.30 ± 5.89%) and P11 to P15 (38.71 ± 6.49%) age groups compared to P16 to P21 (11.47 ± 2.8%). In contrast, GFP expression in MNTB neurons was more prevalent in P16 to P21 (32.86 ± 7.38) compared with the two younger age groups (P6 to P10, 10.37 ± 2.67% and P11 to P15, 10.31 ± 3.02%, respectively). Data were obtained from at least three animals per age group.

**Figure 6:**
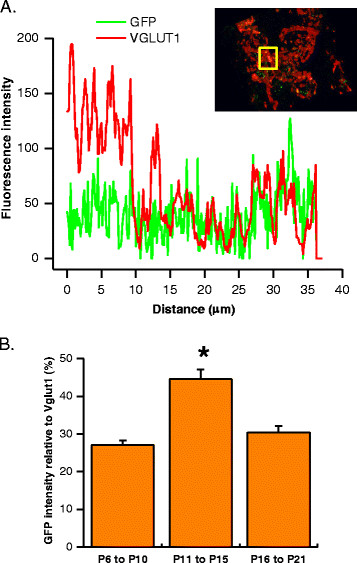
**Development regulation of GFP expression in calyx of Held synapse. (A)** Example of fluorescence intensity profile analysis used to determine relative fluorescence GFP intensity versus the synaptic marker, Vglut1 fluorescence intensity as determined from merge images indicating co-localization of GFP and Vglut1 (insert, yellow square). **(B)** Percent GFP expression from three age groups was analyzed: P6 to P10 (pre-hearing); P11 to P15 (intermediate); and P16 to P21 (post-hearing). Note that GFP intensity is more robust at P11 to P15 and the relative amount is approximately similar between pre- and post-hearing age groups. Data represents mean ± s.e.m. Asterisks indicate significant difference at p < 0.05 (ANOVA) using ORIGIN 7.5 (Northampton, MA) and comparisons were made against P6 to P10 groups. P6 to P10 (n = 151); P11 to P15 (n = 145); and P16 to P21 (n = 103) cells taken from images as described in Figure 5 from at least three animals per age group. Images were acquired as described in *Methods*.

Relative GFP expression in the terminals was further quantified by measuring fluorescence intensity of both GFP and Vglut1 in the calyx as shown in Figure 6A. Total fluorescence intensity from each protein was calculated to derive the ratio of relative intensity between two fluorophores. We found that GFP expression in the presynaptic terminals of P11 to P15 animals (44.57 ± 2.57%) was significantly different from either the younger (27.07 ± 1.15%) or the older (30.42 ± 1.72) (Figure 6B). Taken together, our results demonstrated that GFP expression levels at the calyx of Held-MNTB synapse are dynamically regulated, being more prominent in presynaptic element during early postnatal stages (<P15) but down-regulated at later postnatal stage (>P15), whereas GFP expression in postsynaptic elements becoming increasingly prevalent at later postnatal stages.

## Discussion

The primary goal of this study is to establish a technique that would allow manipulations of genes in the auditory brainstem and other hindbrain neurons, and potentially use this method to understand molecular underpinnings of morphological and functional plasticity of model synapses during normal development and diseases. Previously, *in utero* electroporation-mediated gene delivery method has been utilized to study structures in cortex, retina, thalamus and hypothalamus [[3]–[5]]. However, to our best knowledge, an efficient gene delivery method targeting the hindbrain particularly the auditory brainstem has not been explored. In this work, we established *in utero* injection and electroporation technique targeting subsets of the hindbrain neurons by incorporating both Krox20 construct and region-specific injection.

We have shown that by delivering Krox20 B enhancer and EGFP reporter construct into the third ventricle of embryonic E12 to E13.5 mouse, robust expression of GFP in brainstem and cerebellum (e.g. Purkinje and granule cells) was observed. We explored the potential utility of this technique in synaptic physiology with particular emphasis on the calyx of Held-MNTB synapse. We demonstrated that the distribution and level of GFP expression in the MNTB principal neurons and the calyx of Held vary during development (Figure 4, Figure 5 and Figure 6). On all three stages of development used in this study, fraction of cells expressing GFP on both MNTB and calyx showed no significant difference. However, in pre-hearing and intermediate animals, GFP expression is predominantly in the calyx. Conversely, post-hearing mice showed more expression in MNTB neurons. From these results, we can infer the usefulness of *in utero* electroporation technique in manipulating genes in the presynaptic terminal, and/or the postsynaptic neuron under visual cues of GFP fluorescence in either element or both. For example, rescue experiments such as overexpression of wildtype genes encoding for specific synaptic proteins in genetically knockout or mutant mouse lines can be used to assess their respective role(s) in presynaptic vesicle fusion and exocytosis by using P6 to P15 animals. On the other hand, older mice i.e. P16 to P21 can be used in experiments requiring investigation on the role of a particular protein in postsynaptic neurons. Conversely, one can also utilized this technique to perform gene knockdown or knockout by electroporating siRNA or dominant negative constructs. Alternatively, this technique can also be applied to investigate gene function in spatial and/or temporal manner by incorporating tamoxifen-inducible *Cre* recombinases in Krox20 B enhancer construct that can be transiently expressed. Previous studies have used such a strategy to specifically knockout presynaptic proteins (i.e. RIM) with the Krox20 driver mouse to target the calyx of Held-MNTB synapse [[24]], but our result showing significant GFP expression in the postsynaptic element after P15 raises the caution of its specificity and developmental dependence. We suggest that i*n utero* electroporation method presents an alternative opportunity for variety of genetic manipulations other than stereotactic surgeries in postnatal mice, in which mechanic damage to brain tissues is inevitable. It is however important to note that embryonic survival rate is low when first learning the technique but with practice it rises to 50%. It is also important to consider performing the procedure with minimal stress to both mother and litter to avoid pre-term abortion.

## Conclusions

Overall, we established a relatively efficient and regional and enhancer-specific gene delivery in hindbrain with emphasis on auditory brainstem via *in utero* electroporation technique. This method would potentially allow target-specific knock-down, knock-in as well as rescue experiments to unravel critical molecular substrates underpinning functional and morphological remodeling of synapses during early development and diseases.

## Methods

### Animals

All animal procedures were performed in accordance with The Hospital for Sick Children Animal Care Committee and Canadian Council on Animal Care guidelines for animal research. Pregnant mice (E12.5-E13.5) were purchased from Charles River Laboratories, Wilmington, MA, USA.

### cDNA constructs

For construction of Krox20 B enhancer/β-globin promoter DNA clone encoding GFP in hindbrain structures, the CMV promoter region of pEFGP-C1 vector (Clontech, Invitrogen) was excised and replaced with Krox20 B enhancer and β-globin promoter from cB-LacZ construct kindly donated by Dr. Pascale G-Heberstreit (INSERM, France). Briefly, the ~650 base pairs Krox20 B enhancer/β-globin promoter region of cB-LacZ construct was amplified with Elongase enzyme (Invitrogen) using the oligonucleotides CB1GKrox-AseI: 5′ CGT CAT ATT AAT CCT GCA GGC GGC CGC 3′ and CB1GKrox20-NheI: 5′ C ATA TAT GCT AGC GGC GCC GCG CTC TGC 3′. Polymerase chain reaction (PCR) products were then run in 0.8% agarose gel, excised, purified and subcloned in pGEMT-easy vector and subsequently grown on agar plates for blue/white screening. Selected white colonies were grown into LB media and plasmid DNA from each culture was digested with *AseI* and *NheI* and run in 0.8% agarose gel to confirm positive cloning. The Krox20 B enhancer/β-globin promoter *AseI/NheI* fragment was excised, purified and moved to pEFGP-C1 vector to create the CB-Krox20-GFP construct (Figure 1). This clone was subsequently used for injection into the mouse embryo. All plasmid sequences were confirmed via DNA sequencing.

### *In utero* electroporation

Efficient gene delivery to murine hindbrain was performed using pregnant CD1 mice (Figure 2). Pregnant CD1 mouse (E12-E13.5) was anaesthetized by halothane and a small abdominal midline incision was made. Uterine horns were carefully pulled out onto 37°C pre-warmed saline-moistened cotton gauze, which is placed around the wound. Starting with one horn of the uterus, CB-Krox20-GFP DNA construct or pCDNA3.1 (−) (~1 to 2 μg) mixed with 0.05% trypan blue (Sigma, St. Louis. MO) was injected by air pressure into the lateral ventricles using a mouth-controlled micropipette (Drummond Scientific, Broomall, PA, USA). The tip of the micropipette was directed towards the third ventricle to target hindbrain structures particularly the brainstem. The embryonic brain vesicles are open at early embryonic stages such that DNA injected into the lateral ventricles will flow into the third ventricle. After DNA injection, platinum tweezer-type electrodes (5 mm diameter, Protech, Boerne, Texas, USA) briefly soaked in saline were softly placed on the target region, and five 40 V square of 50 millisecond duration with 950 millisecond intervals were applied by using CUY21EDIT (NepaGene, Chiba, Japan). After electroporation, the uterine horns were carefully replaced back into the abdomen and the wound was closed using sterile surgical 4.0 CV-22 taper sutures (Covidien, Mansfield, MA, USA). Shorter surgical time is preferred as it will yield better viability (<30 minutes per mother). Anafen (10 mg/mL) was injected subcutaneously for post-operative pain and the surgical mother was allowed to recover in a heating pad.

### Brain slices preparation

Brainstem slices were prepared from P6 to P21 mice electroporated with CB-Krox20-GFP DNA construct. Mice were decapitated with small guillotine and brains were immediately immersed into semi-frozen modified artificial cerebral spinal fluid (ACSF) containing the following (in mM): 125 NaCl, 2.5 KCl, 10 glucose, 1.25 NaH_2_PO_4_, 2 Na-pyruvate, 3 *myo*-inositol, 0.5 ascorbic acid, 26 NaHCO_3_, 3 MgCl_2_ and 0.1 CaCl_2_ at a pH of 7.3 when oxygenated (95% O_2_ and 5% CO_2_). Transverse slices of the auditory brainstem containing the medial nucleus of the trapezoid body (MNTB) were cut at a thickness of 100–200 μm using a microtome (VT1000S:Leica, Nussloch, Germany) followed by incubation at 37°C in ACSF containing the following (in mM): 125 NaCl, 2.5 KCl, 10 glucose, 1.25 NaH_2_PO_4_, 2 Na-pyruvate, 3 *myo*-inositol, 0.5 ascorbic acid, 26 NaHCO_3_, 1 MgCl_2_ and 2 CaCl_2_ at pH of 7.3 when oxygenated (95% O_2_ and 5% CO_2_) for 1 hour before experimentation.

Whole brain sagittal and transverse slices were also prepared to determine whether GFP was exclusively expressed within the hindbrain (brainstem and cerebellum) or expression extends towards the forebrain (e.g. cortex and hippocampus). Electroporated mice were anaesthesized using sodium pentobarbital 40 μg per gram body weight administered intraperitoneally. Brains were washed with 1X phosphate buffered saline (PBS) via intra-cardiac perfusion and subsequently fixed by perfusing with 4% paraformaldehyde. Transverse and sagittal brain slices were cut at a thickness of 50 μm using microtome (VT1000S: Leica, Nussloch, Germany).

### Immunohistochemistry and image acquisition

Brainstem slices were fixed in 4% paraformaldehyde in PBS for 30 minutes then rinsed three times with PBS. Slices were then permeabilized in 0.2% Triton X-100 and rinsed three times. The synaptic marker, vesicular glutamate transporter-1 (Vglut1) (Millipore, Temecula, CA, USA) was used to label presynaptic terminals. Following permeabilization, slices were incubated in guinea pig anti-Vglut1 (1:750) overnight at room temperature with gentle agitation. Slices were then washed with PBS prior to incubation in secondary antibody conjugated to Cy3 (1:500; Goat anti-Guinea Pig, Jackson Laboratories, West Grove, PA, USA). After several rinses with PBS, slices were mounted using Vectashield (Vector Laboratories, Burlingame, CA, USA). Fixed transverse and sagittal brain sections were mounted using Vectashield mixed with 4′, 6-diamidino-2-phenylindole (DAPI) to stain for nuclei.

Confocal images and z stack images were acquired with a Zeiss LSM 510 Multiphoton Laser Scanning Microscope equipped with, 405, 488 and 514 nm argon laser lines to visualize nuclei (DAPI), GFP, and Cy3, respectively. Confocal scans were acquired using a 63X (N.A. 1.4) oil objective and the appropriate dichroic filters (MBS 488 and MBS 514). 3D images were rendered using Volocity software (Perkin Elmer, USA).

### Data analysis

Three age groups were analyzed: P6 to P10 (pre-hearing); P11 to P15 (intermediate); and P16 to P21 (post-hearing). Relative fraction of cells expressing GFP from MNTB or calyx alone or from both MNTB and calyx were calculated from at least 14 images per age group. Relative expression of GFP driven by Krox20 B enhancer in the presynaptic terminal was obtained by determining the ratio of the fluorescence intensity of GFP and Vglut1 from a minimum of 100 cells from each age group as mentioned above. All graphs and statistical analyses were performed using ORIGIN 7.5 (Northampton, MA). Data represents mean ± s.e.m. and statistical difference were estimated at p < 0.05 (ANOVA) from a minimum of three animals per age group.

## Abbreviations

DAPI: 4′, 6-diamidino-2-phenylindole

GFP: Green fluorescent protein

LSO: Lateral superior olive

MNTB: Medial nuclei of the trapezoid body

PBS: Phosphate buffered saline

SCA: Spinal cerebellar ataxias

Vglut1: Vesicular glutamate transporter 1

VCN: Ventral cochlear nuclei

## Competing interests

The authors declare that they have no competing interests.

## Authors’ contributions

LSD conceived the project, performed *in utero* electroporation, molecular biology immunohistochemistry, confocal microscopy, data analysis and wrote the manuscript. JA performed *in utero* electroporation. LSL contributed to confocal microscopy. LYW conceived and supervised the project as well as edited the manuscript. All authors read and approved the final manuscript.

## References

[B1] Kolk SM de Mooij-Malsen AJ Martens GJ Spatiotemporal molecular approach of *in utero* electroporation to functionally decipher endophenotypes in neurodevelopmental disorders Front Mol Neurosci 2011 4 37 10.3389/fnmol.2011.00037 22065947 PMC3206543

[B2] Shimogori T Ogawa M Gene application with *in utero* electroporation in mouse embryonic brain Develop Growth Differ 2008 50 499 506 10.1111/j.1440-169X.2008.01045.x 18482402

[B3] Dixit R Lu F Cantrup R Gruenig N Langevin LM Kurrasch DM Schuurmans C Efficient gene delivery into multiple CNS territories using *in utero* electroporation J Vis Exp 2011 ᅟ ᅟ doi:10.3791/2957 10.3791/2957 PMC3197065 21730943

[B4] Matsui A, Yoshida AC, Kubota M, Ogawa M, Shimogori T: **Mouse*****in utero*****electroporation: controlled spatiotemporal gene transfection.** *J Vis Exp* 2011, doi:10.3791/3024. 10.3791/3024 PMC3217635 21860382

[B5] Petros TJ, Rebsam A, Mason CA: ***In utero*****and ex vivo electroporation for gene expression in mouse retinal ganglion cells.** *J Vis Exp* 2009, doi:10.3791/1333. 10.3791/1333 PMC3142865 19779401

[B6] Nishiyama J Hayashi Y Nomura T Miura E Kakegawa W Yuzaki M Selective and regulated gene expression in murine Purkinje cells by *in utero* electroporation Eur J Neurosci 2012 36 2867 2876 10.1111/j.1460-9568.2012.08203.x 22775058

[B7] Fraser S Keynes R Lumsden A Segmentation in the chick embryo hindbrain is defined by cell lineage restrictions Nature 1990 344 431 435 10.1038/344431a0 2320110

[B8] Giudicelli F Taillebourg E Charnay P Gilardi-Hebenstreit P Krox-20 patterns the hindbrain through both cell-autonomous and non cell-autonomous mechanisms Genes Dev 2001 15 567 580 10.1101/gad.189801 11238377 PMC312642

[B9] Lumsden A The cellular basis of segmentation in the developing hindbrain Trends Neurosci 1990 13 329 335 10.1016/0166-2236(90)90144-Y 1699318

[B10] Lumsden A Krumlauf R Patterning the vertebrate neuraxis Science 1996 274 1109 1115 10.1126/science.274.5290.1109 8895453

[B11] Millen KJ Gleeson JG Cerebellar development and disease Curr Opin Neurobiol 2008 18 12 19 10.1016/j.conb.2008.05.010 18513948 PMC2474776

[B12] Oh AK Genetics of vestibulopathy and migraine Curr Opin Otolaryngol Head Neck Surg 2013 21 469 472 10.1097/MOO.0b013e32836464d5 23892793

[B13] Requena T Espinosa-Sanchez JM Lopez-Escamez JA Genetics of dizziness: cerebellar and vestibular disorders Curr Opin Neurol 2014 27 98 104 10.1097/WCO.0000000000000053 24275721

[B14] Voiculescu O Charnay P Schneider-Maunoury S Expression pattern of a Krox-20/Cre knock-in allele in the developing hindbrain, bones, and peripheral nervous system Genesis 2000 26 123 126 10.1002/(SICI)1526-968X(200002)26:2<123::AID-GENE7>3.0.CO;2-O 10686605

[B15] Irving C Nieto MA DasGupta R Charnay P Wilkinson DG Progressive spatial restriction of Sek-1 and Krox-20 gene expression during hindbrain segmentation Dev Biol 1996 173 26 38 10.1006/dbio.1996.0004 8575627

[B16] Schneider-Maunoury S Topilko P Seitandou T Levi G Cohen-Tannoudji M Pournin S Babinet C Charnay P Disruption of Krox-20 results in alteration of rhombomeres 3 and 5 in the developing hindbrain Cell 1993 75 1199 1214 10.1016/0092-8674(93)90329-O 7903221

[B17] Wilkinson DG Bhatt S Chavrier P Bravo R Charnay P Segment-specific expression of a zinc-finger gene in the developing nervous system of the mouse Nature 1989 337 461 464 10.1038/337461a0 2915691

[B18] De S Shuler CF Turman JE Jr The ontogeny of Krox-20 expression in brainstem and cerebellar neurons J Chem Neuroanat 2003 25 213 226 10.1016/S0891-0618(03)00011-5 12706208

[B19] Chomette D Frain M Cereghini S Charnay P Ghislain J Krox20 hindbrain *cis*-regulatory landscape: interplay between multiple long-range initiation and autoregulatory elements Development 2006 133 1253 1262 10.1242/dev.02289 16495311

[B20] Borst JG Soria van Hoeve J The calyx of held synapse: from model synapse to auditory relay Annu Rev Physiol 2012 74 199 224 10.1146/annurev-physiol-020911-153236 22035348

[B21] Wang LY Fedchyshyn MJ Yang YM Action potential evoked transmitter release in central synapses: insights from the developing calyx of Held Mol Brain 2009 2 36 10.1186/1756-6606-2-36 19939269 PMC2794261

[B22] Wimmer VC Nevian T Kuner T Targeted in vivo expression of proteins in the calyx of Held Pflugers Arch 2004 449 319 333 15452710 10.1007/s00424-004-1327-9

[B23] Young SM Jr Neher E Synaptotagmin has an essential function in synaptic vesicle positioning for synchronous release in addition to its role as a calcium sensor Neuron 2009 63 482 496 10.1016/j.neuron.2009.07.028 19709630

[B24] Han Y Kaeser PS Sudhof TC Schneggenburger R RIM determines Ca^2+^ channel density and vesicle docking at the presynaptic active zone Neuron 2011 69 304 316 10.1016/j.neuron.2010.12.014 21262468 PMC3259453

